# Addressing Known hypertensive disOrders of pregnancy in woMen of African descent in Canada (AKOMA): protocol for a mixed method study

**DOI:** 10.3389/fcvm.2024.1471199

**Published:** 2024-12-16

**Authors:** Deborah Baiden, Kara Nerenberg, Edith M. Hillan, Maman Joyce Dogba, Monica Parry

**Affiliations:** ^1^ Lawrence Bloomberg Faculty of Nursing, University of Toronto, Toronto, ON, Canada; ^2^ Departments of Medicine, Obstetrics & Gynecology, Community Health Sciences, University of Calgary, Calgary, AB, Canada; ^3^ Department of Family and Emergency Medicine, Université Laval, Québec City, QC, Canada

**Keywords:** hypertensive disorders of pregnancy, women of African descent, Canada, protocol, mixed-method study, cardiovascular risk

## Abstract

**Background:**

Hypertensive disorders of pregnancy (HDP) predispose a woman to maternity-related cardiovascular morbidity and mortality. However, there is limited literature on HDP among women of African descent in Canada.

**Methods and design:**

A convergent mixed-method study will be used to investigate the intersection of self-reported HDP risks in women of African descent in Canada with a history of a HDP (quantitative, cross-sectional survey) and explore the perception and experiences of women of African descent living in Canada with a history of a HDP in relation to the intersection of risk factors (critical qualitative inquiry, interviews). Quantitative analysis will use SPSS V. 27.0 and thematic analysis will be conducted using NVivo V. 12. A joint display will be used to combine the quantitative and qualitative results.

**Discussion:**

Through the lens of intersectionality, the proposed study aims to provide a comprehensive understanding of the intersection of risks for HDP among women of African descent in Canada with a history of HDP. Furthermore, results could inform future strategies to reduce cardiovascular disease risks.

**Trial Registration Number:**

This is registered in the Open Sciences Framework as follows: https://doi.org/10.17605/OSF.IO/R6CKY.

## 1 Introduction

The classification of hypertensive disorders of pregnancy (HDP) includes hypertension prior to pregnancy, gestational hypertension, preeclampsia, and added hypertensive effects (1). Added hypertensive effects of HDP include signs and symptoms of organ damage or injury in addition to an elevated blood pressure, such as newly discovered or increased protein levels in the urine (1). Hypertension prior to pregnancy is defined as hypertension before 20 weeks of pregnancy, gestational hypertension is defined as hypertension at or after 20 weeks of pregnancy, and preeclampsia is gestational hypertension with organ system effects (1). Race and ethnic differences related to HDP accounts for the high risk of HDP mortality and morbidity in women of African descent (2). Black women in the United States are approximately 3.6 times more likely to die from pregnancy-related complications and about five times more likely to die from HDP and other cardiovascular complications in comparison to White women (3). Similarly, in the United Kingdom, Black women have a four times higher risk of dying from pregnancy-related complications including HDP (4). Women of African descent in Finland have a higher risk of preeclampsia in comparison to White women of Finnish origin (5). There is compelling evidence on the high risk of maternity related cardiovascular complications including HDP among women of African descent yet there is inadequate literature on this population in Canada. There is also immense literature on HDP in White women living in North America and Europe in comparison to Black women who are at a higher risk of HDP (6). The dearth of HDP literature in women of African descent shows the need for more research on HDP with this population in a way that centers their voice and contributions. Engaging women of African descent as patient partners is one important way of centering their voices and lived experiences in HDP research. It is also crucial to involve women of African descent as patient partners in research towards leading the change in reducing the burden of HDP in populations of African descent and informing equitable HDP research. The study has the acronym of AKOMA, which means heart in Akan/Twi, a language spoken across West African countries. This acronym pays homage to the “motherland” (Africa), out of which populations of African descent derive their cultural heritage and being.

The health equalities experienced by populations impact on their health outcomes. The COVID-19 pandemic put the spotlight on the disproportionate impact on communities of African descent and other populations experiencing marginalization (7). In addition, the death of pediatric chief resident, Dr. Chaniece Wallace through preeclampsia showed that socioeconomic status was not the only risk factor (8). In Canada, people of African descent experience health inequalities, which leads to worse health outcomes for this population in comparison to other populations (9). The United Nations' Working Group of Experts on People of African Descent advocated for transformative measures such as the use of intersectionality in the framing of work to address disparities among populations of African descent globally (10).

For this study, the term “African descent” refers to people who identify as African, African Canadian, Black Canadian, Afro-Caribbean, African American, Afro-Indigenous, and Afro-Latina. The United Nations declared 2015 to 2024 as the International Decade of the People of African Descent. People of African descent live not only in the African continent but have also been dispersed outside the African continent through the trans-Atlantic slave trade and afterwards through voluntary migration. The term “Diaspora” will refer to the population of African descent who live outside the African continent by way of the consequences of the slave trade or through migration. In 2019, the United Nations' Educational, Scientific, and Cultural Organization (UNESCO) declared 24th January as the World Day for African and Afro-descendant Culture to celebrate and in recognition of the cultural diversity of people in the African continent and the Diasporas globally (11).

About 275 million women worldwide were diagnosed with cardiovascular diseases in 2019 (12). In recent years, the global rates by which women died from cardiovascular diseases has experienced a standstill (12). There is a high burden of cardiovascular disease in women in low-income countries, including countries in the Sub-Sahara African region (12). Nevertheless, women of African descent in high income countries including Canada and the US, are at high risk of maternity-related cardiovascular diseases such as HDP (13). Further, women of African descent in Canada who are low-income earners have a higher risk of hypertension and diabetes in comparison to White Canadians (14). As of 2016, there are approximately 1.2 million Black people in Canada (15). Almost 52% of the Black population in Canada are women and 285,130 of the Black population in Canada were born in Africa (16). There is an increase in Black newcomer people migrating from African countries since 2011, up from 4.8% prior to 1981 to 65.1% between 2011 and 2016 (16). In comparison to the United States and the United Kingdom, there is a paucity of health data on women of African descent in Canada. There is the need for a broader understanding of the cultural and structural nuances of cardiovascular disease risks and the intersection of the social identities of women of African descent with known HDP. This is significant because Canada has a multicultural and diverse population, and it is important to factor culture and intersectionality in healthcare for health equity. More importantly, the population of women of African descent in Canada is growing and by the year 2036, will increase by up to 5.6% (16). This study will also provide an avenue for women of African descent to discuss and contribute to cardiovascular health, particularly HDP research and consequently cardiovascular health care. Moreover, this study will engage a woman of African descent as a patient partner in HDP research, centering the voice of a person from a marginalized population at risk yet underrepresented in HDP research. Cardiovascular disease risks in women of African descent should be explored to improve cardiovascular health outcomes (12, 17). Findings from this study could begin to address an informed approach to culturally appropriate care and intervention research aimed to reduce disparities in maternity-related cardiovascular outcomes among women of African descent in Canada.

The overall aim of this proposed study is to provide a comprehensive understanding of the intersection of risks for HDP in women of African descent in Canada. Specific objectives include: (1) determining the intersectionality of self-reported risk factors for HDP in women of African descent in Canada with a history of HDP (quantitative, cross-sectional survey), and (2) exploring the perceptions and experiences of the intersection of risks for HDP in women of African descent in Canada following a HDP (critical qualitative inquiry, interviews).

## 2 Methods

### 2.1 Design

A convergent mixed methods approach will be used for this study. A mixed methods approach combines the benefits of generalization from the quantitative methods and captures the richness and depth of personal experiences and understanding from qualitative methods. Hence, a convergent mixed methods approach is appropriate for achieving the study's purpose of providing a comprehensive understanding of the intersection of risks for HDP among women of African descent in Canada with a history of HDP. The study consists of a cross-sectional survey to investigate the intersection of self-reported HDP risks in women of African descent in Canada and a critical qualitative inquiry to explore the perception and experiences of women of African descent living in Canada with a history of a HDP, in relation to the intersection of risk factors.

### 2.2 Study frameworks

The theory of intersectionality will be used to guide this study as it assists to explain the individual and societal identities (e.g., socioeconomic, and structural determinants) that influence disparities in maternal health outcomes and health care service utilization among women of African descent (18). Also, it is essential to study intersectionality in risk projection and outcomes of cardiovascular disease (19, 20).

The Strategy for Patient-Oriented Research (SPOR) Patient Engagement Framework (21) will also be used to guide this study. A woman of African descent will be engaged as a patient partner in research and will be involved from the framing of data collection tools to the dissemination of findings. Patient engagement of a woman of African descent is supported by the United Nations' Sustainable Development Goal 5 aiming to attain gender equality. A patient partner will be trained and supported to engage in research process and interpretation. Knowledge generation will be a collective effort of equal power among research team members and the patient partner, with the patient partner: (1) Informing consent language for the Research Ethics Board application; (2) Reviewing the survey and interview guide and supporting recruitment efforts for the study; (3) Contributing to the merging and interpretation of study results; and (4) Involved in publication and presentations. The Guidance for Reporting Involvement of Patients and the Public (22, 23) (GRIPP 2 short form) will guide the reporting of patient engagement (Supplementary Table S1).

### 2.3 Eligibility criteria

This mixed-method study will be conducted across all provinces and territories in Canada. This study will use the same sample for both the quantitative and qualitative strands. Women who will be eligible for this study include those who: (1) self-identify as being of African descent; (2) reside in Canada; (3) have a history of HDP within the past ten years; (4) are 18 years and older; and 5) can effectively communicate in the English language. Women of African descent within a ten-year range will be eligible since the evidence suggests that a history of HDP predisposes a woman to future cardiovascular morbidity and mortality of up to ten years (24). Potential participants who are pregnant at the time of the mixed-method study will not be included as the focus of this study is on intersection of self-reported risks for HDP and the perception and experiences of HDP risks in women of African descent.

### 2.4 Recruitment

Broad recruitment strategies will be used to reach participants across all provinces and territories in Canada. Flyers with study information including the purpose of the study, eligibility criteria and contact information will be used. A digital version of the flyer will also be used for distribution on social media channels/groups of community-based organizations of African descent. Flyers will be posted on the bulletin boards of faith-based centers with a predominant congregation of African-descent, hair salons serving clientele of African descent, community health centers, and community-based organizations of African descent in Canada. Furthermore, eligible participants will be encouraged to inform potential participants in their networks by word of mouth. Permission will be sought for recruitment from the various health care facilities and organizations. The use of these broad recruitment strategies will be effective in obtaining the needed sample. Cudjoe and colleagues (25) engaged African immigrant churches for recruitment of women of African descent in their study on the cervical screening habits of African immigrant women in the United States. A total of 167 women of African descent were recruited within a one-year period (25). A total of 355 Black women in the United States were recruited for a cross-sectional survey within a four-month period (26). Additionally, religious leaders of African descent and hairdressers in Black salons will be contacted to help in recruitment. These religious leaders and hairdressers are gatekeepers in the community and can assist in accessing this population. Gatekeepers in research includes individuals, stakeholders, and group representatives that primarily link researchers to eligible participants based on their understanding and belonging to the community (27). Thus, these gatekeepers would provide access to the community of African descent as well as serve as valuable resources for recruitment. Furthermore, the use of casual conversations as a recruitment and engagement strategy when conducting research with populations of African descent is recognized to be successful (23, 28). Therefore, the use of sampling methods that encourage eligible women of African descent to recruit from their social networks would be employed. An honorarium of a $10 gift card would be given to honor the time spent in participation and contribution to the study. The gift card will be given for participation in each arm of the study as a token of appreciation. The compensation for patient partners will follow the SPOR guidelines (21). These recruitment strategies could help mitigate the challenge of hesitancy of women of African descent to participate in health research. Inadequate representation of ethnic minorities in health research (29) impacts generalizability of research results, health equity, and ethnic specific health care services (30).

### 2.5 Quantitative Arm

A cross-sectional survey design will be used for this arm of the study. The cross-sectional survey design is appropriate to collect relevant descriptive data that will be used to make associations (31). Furthermore, this design is time and cost effective. The Strengthening the Reporting of Observational Studies in Epidemiology (STROBE) guidelines for reporting cross-sectional studies will be followed (32).

#### 2.5.1 Participants

Participants eligible for this study will: (1) self-identify as being of African descent; (2) reside in Canada; (3) have a history of HDP within the past ten years; (4) be 18 years and older; and (5) be able to effectively communicate in the English language.

#### 2.5.2 Variables

Demographic and risks for HDP variables will be collected in the survey as determined from our recent scoping review (20). Variables will include age, gender (identity, roles, relations, institutionalized) (33), immigration status, length of time in Canada, and cultural background (i.e., African, African Canadian, Afro-Caribbean, African American, Afro-Latina, Afro-Indigenous, Black Canadian). Data will also be collected on pregestational weight, discrimination, and acculturation. A database will be created in Microsoft Excel. Data will be entered, cleaned, and analyzed imported into the Statistical Package for the Social Sciences (SPSS) version 27.0 for analyses (34).

#### 2.5.3 Data sources/measurement

The four themes of HDP risks derived from our recent scoping review (20): (1) biological risks, (2) individual traditional risks, (3) race and ethnicity, geographical location, and immigration status, and (4) gender-related risks will inform the survey variables. The Genesis-Praxy Gender Questionnaire (35) will be adapted and used to guide the collection of data on gender variables. The gender variables will also be informed by a more recent review of gender-related variables that include discrimination (35). Self-reported measures will be used to measure respondents' risks for HDP. As this study is patient-oriented, a patient partner will be engaged in survey development with the research team. The Vancouver Index of Acculturation will be used to measure acculturation (36). The survey will first be pilot tested with 10 respondents (37) after ethical approval of the study. The main purpose of this pilot testing is to determine practicality of methods (38).

#### 2.5.4 Sample size

Using an estimated prevalence of about 9% for HDP in Black women in the United States (39), a calculation of the sample size was estimated using 9% of the total population of Black women living in Canada, which is approximately 620,000 (16). The sample size for this arm of the study is an estimated at 382 women of African descent, using a 95% confidence level, a margin of error of 5%, and an estimated patient population size of 55,800 women of African descent. The sample size of 382 women was estimated using an online sample size calculator (Qualtrics®) (40).

#### 2.5.5 Data collection

Participants will have access to the surveys after determination of eligibility by the researcher (41). Research Electronic Data Capture (REDCap) (42, 43) will be used for online survey distribution and paper-based surveys will be used for those interested participants who may not have access to reliable internet connection. Participants will have access to the surveys after determination of eligibility by the researcher or the seeds (41). The response burden of the survey will be kept as low as possible to also help with getting the survey back fully completed.

#### 2.5.6 Statistical analyses

Univariate statistics will be used to describe the demographics of the sample, including frequencies, percentages, means and standard deviations. Missing data will be assessed and if excessive (i.e., >10%), multiple imputation will be considered. At the individual level, missing data will be prevented by pilot testing the survey to identify pitfalls and ensuring that the number of items on the survey are not too many for respondents to lose interest (44). In addition, questions on key variables will be made mandatory. The dichotomized dependent variable is the history of HDP (0 to <6 years and ≥6 to 10 years). A two-stage analytical strategy (45, 46) will be undertaken using logistic regression to model history of a HDP 0 to <6 years and ≥6–10 years according to the following axes of difference set *a priori* and guided by four themes: (1) biological risks [low Vitamin D (yes, no)], (2) individual traditional risks for HDP (BMI > 25 [yes, no], family history of a HDP [yes, no], diabetes [yes, no], chronic hypertension [yes, no]), (3) geographical location (urban living: population of ≥1,000) [yes, no], poor neighbourhood [neighborhood income below $26,000 (yes, no)], and migration status (migration status >10 years [yes, no], more acculturated [born in Canada and proficient in official languages (yes, no), immigration status documented (yes, no)], as HDP risks, and (4) gender related risks [without spouse/partner (yes, no), income level, education level] for HDP. The first stage of analysis will model main effects of each factor. Unadjusted and adjusted models will be run to determine what axes of difference are associated with a history of HDP 0 to <6 years and ≥6–10 years and whether they are independent of each other. In the second stage of analysis, interaction terms will be added to each model and Akaike's Information Criterion (AIC) will be used to determine if the model containing the interactions provides a better fit to the data than the main effects model. A better fitting model would provide evidence that at least some of the axes of differences tested have a multiplicative effect. If the model with the interaction is found to be a better fit to the data, the significance of the coefficients of each interaction term will be evaluated. A *p*-value of ≤0.05 will be considered statistically significant. All *p*-values that will be reported for this study will be two-tailed. To ensure bot reduction in survey completion, the survey link will not be shared on publicly accessible social media platforms, survey will include free-form text data, and a CAPTCHA (47).

### 2.6 Qualitative Arm

Critical qualitative inquiry (48) will be used. Critical qualitative inquiry is qualitative research in the transformative lens that aims to reveal marginalization and create change by centering the voices of the marginalized using inquiry (49). Critical qualitative inquiry will be used to center the voices of women of African descent as they describe their perception and experiences in relation to the intersection of risk factors for HDP. The critical qualitative inquiry will be appropriate for this study because it is situated in the transformative lens (48). The Consolidated criteria for reporting qualitative research (COREQ) will be followed in reporting this study (50).

#### 2.6.1 Participants

Participants will be selected using purposive sampling. Women who completed the survey and are willing to participate in an interview will be purposefully invited. Purposive sampling is appropriate in selecting participants that will provide information to best answer the research question (51).

#### 2.6.2 Sample size

A sample size of 15–20 participants will be included for this study. This sample size will be sought because critical qualitative inquiries have been conducted using this range to data saturation (52).

#### 2.6.3 Setting

The interviews will be conducted in-person, virtually, or by telephone based on convenience of the participants. Participants will be encouraged to choose a location that is private, quiet, and without the presence of non-participants (53).

#### 2.6.4 Data collection

Qualitative data collection will be done concurrently and independent of the quantitative strand (54). In-depth individual semi-structured interviews to elicit data will be conducted. A semi-structured interview guide will be used to facilitate the interviews and will include intersectional questions (55). Interviews will be conducted in a conversational manner with probes to elicit more information. To build rapport and trust in women of African descent, non-research related conversations such as asking about the well-being of participants and their families will be done prior to the start of the interview (53). The interviews will be audiotaped and are anticipated to last between 45 and 60 min. Recruitment and data collection will continue until no new information could be obtained from additional interviews (51). Transcripts will be sent to participants for feedback and accuracy. A journal will be kept throughout the research process to record field notes and reflections on positionality, power, and intersectional influences in the research process using a social identity map (56). Reflexivity in critical qualitative research using social identity mapping is important because it allows the researcher to critically think about their social identities, unpack their position in a socially constructed hierarchy, and the impact on the research process (56). Also, reflexivity will help to “clarify the bias” that I would be bringing to this study as a researcher (54). Recruitment for interviews will end after data saturation has been achieved, and no new data is being elicited (51).

#### 2.6.5 Data analysis

Qualitative data analysis will be completed using an intersectional lens to capture a multidimensional understanding (55) of perceived risk factors for HDP by women of African descent. This type of analysis is a two-step approach and will first involve a thematic analysis to identify themes and patterns and then another analysis completed using intersectionality to unravel multidimensional interpretations of the themes and patterns identified (55). NVivo data analysis software (57) will be used to organize and facilitate analysis. One follow-up interview will be conducted to cross-check significant survey results and themes with participants for comments on accuracy (54). A patient partner will be engaged in the analysis and confirmation of themes. Co-authors will also be involved in data analysis through discussions on the potential themes.

### 2.7 Integration of findings

A joint display table will be used to practically merge findings in a single illustration, this would be followed by a discussion and interpretation of the results. The integration of mixed methods research through merging with a joint display table allows for side by side analysis and juxtaposition of findings (58). Integration through merging of the results is appropriate for this study because it is a convergent design and has parallel concepts in both arms of the research (58). A patient partner will be engaged in merging and interpretation of results.

### 2.8 Ethics

Ethics approval has been obtained from the University of Toronto's Health Sciences Research Ethics Board (REB) (Protocol#: 00043947, March 4th, 2023). Women of African descent who are eligible to participate in the study will be provided with a letter of information and informed consent form in clear and simple English language. The letter of information and consent form will follow guidelines according to the University of Toronto's Health Sciences REB with input from the patient partner to ensure appropriate language and readability. Information on the risks and benefits of partaking in the research will be provided to participants verbally as well as in written form. Women of African descent eligible for the study will be informed that participation is completely voluntary and they could refuse participation, withdraw from the study at any time, or not answer questions without any consequences such as being blocked from accessing health services. The proposal will follow the three core principles (respect for persons, concern for welfare and justice) of the Tri-Council Policy Statement: Ethical Conduct for Research Involving Humans -TCPS2 (59). The principle of respect for persons would acknowledge that women of African descent are not a monolithic group, thus the intersections of their multiple identities will be considered to ensure that they independently make decisions regarding engaging in this study (59). Concern for welfare would recognize the importance of ensuring that participation in the proposed study will not further marginalize this population and that privacy and confidentiality of personal-identifiable data will be protected (59). Justice would ensure that women of African descent are provided with the same information for recruitment, treated equally, and the power differentials between researcher and participants are reduced by including a patient partner of African descent (59).

### 2.9 Strengths and limitations

This study would be one of the first studies to use a mixed methods approach to provide a comprehensive understanding of the intersection of risks for HDP in women of African descent with a history of HDP in Canada. HDP can increase a woman's risk of cardiovascular disease in future (60). This proposed research will provide a unique contribution to the conceptualization of the prevention and management of cardiovascular disease risks in women of African descent in Canada. The proposed framing of the convergent mixed method approach in an intersectional theoretical lens is a strength for the study. The theory of intersectionality recognizes inequalities, marginalization, and cultural influences. This study will engage a woman of African descent as a patient partner in HDP research to centre the voice of a person from a marginalized population, at risk for HDP, and yet underrepresented in research. A limitation of the eligibility criteria is that it may exclude the contribution of non-English speaking women of African descent. However, findings from this study could be applied to all women of African descent living in Canada because language was not found to be an HDP risk factor (20).

## 3 Discussion

Canada, like many other countries, is multicultural and becoming increasingly diverse. This diversity drives the need for tailored and culturally specific health care (61). To the best of our knowledge, this will be one of the first studies to use a mixed methods approach to provide a comprehensive understanding of the intersection of risks for HDP in women of African descent with a history of HDP in Canada. This proposed research will provide a unique contribution to the conceptualization of the prevention and management of cardiovascular disease risks in women of African descent with a history of HDP. Furthermore, the results will provide a valuable explanation for the HDP disparities experienced by women of African descent in Canada. The contribution to knowledge could inform direction for future research such as determining structural factors that impact on cardiovascular health of women of African descent. Moreover, results will inform maternal and public health nurses and other health care professionals in their support, education, and care of women of African descent and as well contribute to discharge planning and culturally competent care. The significance of this proposed study is supported by the United Nations' Sustainable Development Goals number 3 of good health and wellbeing, number 5 of gender equality, number 10 of reduced inequalities in cardiovascular disease, and number 17 of partnerships, with patients and communities in research (62).

## Data Availability

The original contributions presented in the study are included in the article/Supplementary Material, further inquiries can be directed to the corresponding author.
