# Supplementary material for: Addressing Known hypertensive disOrders of pregnancy in woMen of African descent in Canada (AKOMA): protocol for a mixed method study
Source: Front Cardiovasc Med. 2024 Dec 16;11:1471199. doi: 10.3389/fcvm.2024.1471199 (PMC11683087; doi:10.3389/fcvm.2024.1471199)
Supplement: Supplementary file 1 [file Table1.docx]

| Section and Topic | Item | Reported on Page Number |
| --- | --- | --- |
| 1. Aim | Report the aim of patient and public involvement (PPI) in the study | 2 |
| 1. Methods | Provide a clear description of the methods used for PPI in the study | 3, 4, 5, 6 |
| 1. Study Results | Outcomes - Report the results of PPI in the study, including both positive and negative outcomes | Not Applicable |
| 1. Discussions and conclusions | Outcomes - Comment on the extent to which PPI influenced the study overall. Describe positive and negative effects | Not Applicable |
| 1. Reflections/critical perspective | Comment critically on the study, reflecting on the things that went well and those that did not, so others can learn from this experience | Not Applicable |

**Supplementary Table S1** Guidance for Reporting Involvement of Patients and the Public – Short Form (GRIPP 2 – SF).
